# Building Resilient Pediatric Care: Lessons from Service Disruptions for Children with Special Healthcare Needs During the COVID-19 Pandemic in Germany

**DOI:** 10.3390/children13010037

**Published:** 2025-12-26

**Authors:** Lia von Spreckelsen, Anneke Haddad, Shrabon Insan, Henriette Högl, Annette Mund, Thorsten Langer, Anne Geweniger

**Affiliations:** 1 Department of Orthodontics, Medical Center-University of Freiburg, 79106 Freiburg, Germany; 2 Institute for Infection Prevention and Control, Medical Center-University of Freiburg, 79106 Freiburg, Germany; 3 Department of Neuropediatrics and Muscle Disease, Medical Center-University of Freiburg, 79106 Freiburg, Germany

**Keywords:** healthcare provision, COVID-19, children with special healthcare needs, caregivers, crisis-resilient care structures

## Abstract

**Highlights:**

**What are the main findings?**

- During the first round of the survey, up to 88% of children with special healthcare needs (CSHCN) experienced reduced access to pediatric healthcare and therapeutic services.
- Disruptions in care were closely linked to higher disease complexity, while no significant associations were identified with socioeconomic status or caregiver mental health.

**What are the implications of the main findings?**

- The study reveals critical structural weaknesses in pediatric healthcare systems, highlighting the urgent need to strengthen continuity of care for children with complex medical needs.
- Developing crisis-resilient, integrated, and family-centered pediatric healthcare frameworks is essential to protect CSHCN and ensure equitable access during future public health crises.

**Abstract:**

**Introduction:** This study aimed (1) to describe services involved in healthcare provision for children with special healthcare needs (CSHCN) and explore changes in the frequency of service provision reported by parents during the first wave of the COVID-19 pandemic; (2) to analyze associations between healthcare service provision and disease complexity; (3) to explore potential associations of changes in frequency of service provision with disease complexity, socioeconomic status (SES), and psychosocial factors; and (4) to generate actionable insights for building crisis-resilient care systems. **Methods:** A sequential series of cross-sectional online surveys at three points in time was conducted among caregivers of children with and without special healthcare needs in Germany. We analyzed data from the first survey (08/2020–10/2020). **Results:** Among CSHCN, reductions in treatment reached up to 88.4%. Positive associations between the reduction in treatment during the pandemic and disease complexity could be shown. There was no evidence for associations between reductions in healthcare provision, SES, and/or mental health. Structural vulnerabilities within existing care pathways for children with and without special healthcare needs could be identified. **Conclusions:** The findings highlight major gaps in healthcare continuity and underscore the urgent need for crisis-resilient care structures. CSHCN with more complex needs require prioritized, consistent, and structurally protected access to multidisciplinary services. The study calls for long-term investment in integrated, cross-sectoral, and family-centered healthcare frameworks to safeguard CSHCN in future public health emergencies.

## 1. Introduction

After the World Health Organization (WHO) declared the COVID-19 outbreak a global pandemic, worldwide intervention measures were taken to curb the spread of SARS-CoV-2, thus aiming to protect the healthcare system from collapsing [1]. An international WHO survey showed that over 70% of medical–therapeutic care for children had been cancelled worldwide during the first wave of the pandemic (01/2020–06/2020) [2]. Full school closures occurred at a national level among almost all European countries during the pandemic (01/2020–03/2022) [3]. In Germany, the first COVID-19 wave lasted 6 months (01/2020–06/2020) [4]. Since then, extensive data have emerged on how COVID-19 regulations have impacted entire healthcare systems worldwide [5]. While children generally showed only a low primary burden of disease, the impact of disruptions in their everyday life on mental well-being, education, and social and other support systems has been widely reported [2,6]. In this context, children with special healthcare needs are a particularly vulnerable group, as international research relating to the provision of medical care and the physical and mental well-being of CSHCN suggests [5]. Analyses of national survey data and pediatric cohorts in the United States show that CSHCN experienced reduced preventive visits, deferred elective procedures, and difficulty obtaining appointments as services were curtailed or clinics closed. These disruptions led to increased unmet needs for specialist and mental healthcare, particularly among children from racial/ethnic minority groups and those who were uninsured or had coverage gaps [7]. Furthermore, CSHCN faced a widespread interruption in rehabilitation and supportive therapies (e.g., physiotherapy, occupational, speech, behavioral interventions), with some cohorts documenting the complete loss of all therapies for a substantial proportion of children early in the pandemic. The closure of schools eliminated access to school-based health and developmental services and equipment, with very limited transfer of these supports into home or community settings [8].

However, data on healthcare provision during the first wave of the COVID-19 pandemic for CSHCN in Germany are scarce [5,6]. This study therefore had the following aims:

(1) To describe services involved in healthcare provision for CSHCN and explore changes in the frequency of service provision during the first wave of the COVID-19 pandemic;
(2) To analyze associations between complexity of service provision and disease complexity;
(3) To explore potential associations of changes in the frequency of service provision during the first wave of the COVID-19 pandemic and associations with disease complexity, psychosocial factors, and SES;
(4) To generate actionable insights for building and reimagining crisis-resilient pediatric care systems.

By identifying structural weaknesses and population-specific risks, this study offers critical insights for building more resilient, equitable, and inclusive care infrastructures.

## 2. Materials and Methods

**Study design.** A cross-sectional online survey at different points in time since the beginning of the pandemic was conducted among caregivers of children (≤18 years) in Germany. Caregivers of children aged 18 years or younger living in the same household were eligible to participate. For this study, data from the first survey (08/2020–10/2020) were analyzed. Participants, i.e., caregivers of healthy children, caregivers of children with less complex needs (e.g., well-controlled asthma), and caregivers of children with more complex needs (e.g., home ventilation required) were recruited via the patient organization Kindernetzwerk e.V. (Aschaffenburg, Germany), social media, and freely accessible websites.

**Public involvement.** Kindernetzwerk e.V., a large German patient organization for families with CSHCN, was involved in the entire process of developing the study concept and methodology, promoting the study, data collection, and the dissemination of study results.

**Measures.** Disease complexity was assessed using the CSHCN Screener, a standardized five-item parent-reported screening instrument that considers non-disease-specific measures to identify chronic physical, mental, developmental, and/or other health problems [9,10]. The questionnaire asks about five possible health impacts. A child is considered to have a special healthcare need if they (1) are limited in activities that peers can normally do; (2) take prescribed medication (excluding vitamins); (3) receive specialized therapy such as physical, occupational, or speech therapy; (4) require or use extra medical, mental health, or educational services; or (5) receive treatment or counseling for emotional, behavioral, or developmental issues. A maximum score of 5 can be obtained if all items are answered in the affirmative. As suggested by Bethell et al., children were grouped into children without special healthcare needs (CSHCN = 0), children with less complex needs (0 < CSHCN ≤ 2), and children with more complex needs (CSHCN ≥ 3), indicating multiple types of needs [10].

Care complexity was operationalized as the number of medical and therapeutic disciplines involved in a child’s care. Overall care complexity was represented by a composite measure aggregating the reported service utilization across all medical and therapeutic sub-domains (for the precise labeling of the subcategories, see Table 1). To facilitate standardized categorization, all services were equally weighted despite heterogeneity in clinical relevance. For sector-specific analyses, parallel composite measures were constructed for service utilization within the medical sector and the educational sector.

Reductions in service utilization during the first round of the survey were assessed based on parental reports of reduced or discontinued services across the medical, educational, and social care sectors. Composite indicators were created to capture overall reductions in service utilization across sub-domains. Reasons for service reduction were not assessed; therefore, this measure reflects changes in service utilization frequency during the first round of the survey.

The mental well-being of children and adolescents (≤16 years) was evaluated using the parent version of the Strengths and Difficulties Questionnaire (SDQ), an established and validated survey for parent-reported mental health problems in children and adolescents [11]. The validity and reliability of the German version correspond to those of the original English version [11,12]. The total difficulties score ranges from 0 to 40, with high values indicating psychological or behavioral problems [11]. The preschool version of the SDQ varies from the standard parent-reported version in three items that are tailored to younger children (www.sdqinfo.org) [13]. The German adaptation has been validated with 3 to 5 year olds. Reflecting the work of previous studies, age-appropriate SDQ versions were used to assess comparable data [13]. In accordance with published standards, a cut-off value of 13 was set for the total difficulties score [12].

Caregiver mental well-being was assessed using the WHO-5 Well-Being Index (WHO-5). Since the first publication in 1998, various studies have proven its validity and reliability [14]. The WHO-5 assesses self-reported current mental well-being and consists of five statements that are rated on a six-point Likert scale (0 = never, 5 = always). Scaled scores (ranging from 0 to 100) are obtained by multiplying the original score (0–25) by four. High scores indicate good psychological well-being. According to published standards, a cut-off value of 50 can be used for depression screening [14].

Sociodemographic characteristics were assessed using individually drafted items for age and gender, as well as country of birth, marital status, place of residence, household size, education, occupation, and monthly household income. An SES index was constructed to measure socioeconomic status, based on the approach of the Federal Health Survey for Children and Adolescents in Germany (KiGGS) [15]. It comprised the caregivers’ education, job, and income, weighted according to the household size and added up to the total score [15].

**Statistical Methods**. To enable the inclusion of all cases in the statistical analysis (N = 1619), missing values were addressed with regression-based multiple imputation. Assuming that data were missing at random, the expectation–maximization algorithm with Monte Carlo draws produced 5 imputed datasets. Linear regression with predictive mean matching was used for continuous variables and proportional odds regression for ordinal variables. The SDQ items, the five WHO-5 items, and household income plus a set of auxiliary predictors (caregiver’s age and profession, child’s age, CSHCN score) entered the imputation model. Estimates were pooled across imputations and the pooled estimates were used in the final analyses.

For all other relevant variables included in the analysis, percentages of missing values are reported. Descriptive data analysis included absolute and relative frequencies, mean values, and standard deviations. Chi-squared tests were used to determine the level of association between SDQ/WHO-5 scores and CSHCN status. Fisher’s exact test was used to assess associations between healthcare provision in the educational sector and CSHCN. Associations between disease complexity and complexity of healthcare provision, as well as reductions in healthcare provision, were tested using simple linear regression. The multiple linear regression calculation model was used to determine associations between reductions in medical and therapeutic care and disease complexity, SES, and psychosocial factors of both children and caregivers. The analysis was restricted to children aged 3–16 years, in accordance with the SDQ age limit as outlined above. Because the CSHCN Screener incorporates three items that directly assess health service need/use (items 2–4), we conducted a sensitivity analysis removing these items. A revised ‘complexity without use’ score was calculated by summing only items 1 (functional limitation) and 5 (family-reported medical complexity). Statistical analysis was performed using IBM SPSS© Statistics Version 28 (IBM Corporation, Armonk, NY, USA).

## 3. Results

**Participants’ characteristics.** A total number of n = 2004 persons accessed the online survey, of which n = 1619 had children ≤18 years living in the same household, thus meeting the inclusion criteria. Of the n = 385 persons who were not included in the final sample, n = 26 did not meet the inclusion criteria. The remaining n = 359 accessed the survey but did not answer any of the survey questions following consent to participate; hence, no survey data were available from these participants. Table 1 shows the sociodemographic characteristics of the study population. The respondents’ mean age was 41 years (SD = 6.9), 90.1% were female, and 70.4% had completed at least 10 years of schooling, corresponding to a general high school diploma. There were significant differences according to SES and household characteristics between CSHCN and children without special healthcare needs (Table 1). Caregivers of CSHCN were more likely to live in a small town or rural area (57.7% vs. 47.2%), to work part-time (19.5% vs. 13.0%), and to be single parents (10.5% vs. 6.4%). Families with CSHCN are over-represented in the lowest income quintile (28.6% vs. 18.6%) and under-represented in the middle- and high-income quintiles. A larger share of CSHCN families reported a decrease in household income (31.3% vs. 24.6%).

**Table 1:** Sociodemographic characteristics (N = 1619).

			Health Conditions of Children					
	Total		Children Without Special Healthcare Needs		CSHCN			
	M (SD)	Range	M (SD)	Range	M (SD)	Range		
Age in years (N = 1619)								
Caregivers	41.2 (6.94)	17–72	40.8 (6.80)	17–72	41.8 (7.10)	17–61		
Children	8.1 (4.2)	0–18	7.7 (4.1)	1–18	9.0 (4.4)	0–18		
	n	%	n	%	n	%	Chi ^2^	*p*
Gender of respondent (N = 1619)							9.94	0.0016
Male	161	9.9	113	11.9	48	7.2		
Female	1458	90.1	835	88.1	623	92.8		
Gender of child (N = 1619)							1.92	0.38
Male	899	55.5	526	55.5	373	55.6		
Female	710	43.9	414	43.6	296	44.1		
Diverse	10	0.6	8	0.8	2	0.3		
Disease complexity of child (N = 1619)								
0 < CSHCN ≤ 2	193	11.9	-	-	193	28.8		
CSHCN ≥ 3	478	29.5	-	-	478	71.2		
Country of birth of caregivers (N = 1281)							2.47	0.12
Germany	1187	92.7	698	91.7	489	94.0		
Other	94	7.3	63	8.3	31	6.0		
Country of birth of partner (N = 1282)							2.25	0.13
Germany	1129	88.1	667	87.0	462	89.7		
Other	153	11.9	100	13.0	53	10.3		
Place of residence (N = 1619)							17.68	<0.001
City (>100,000 inhabitants)/suburbs	784	48.5	500	52.8	284	42.3		
Small town/rural area	835	51.5	446	47.2	387	57.7		
Relationship status (N = 1619)							11	0.004
With partner, in same household	1441	89.0	863	91.0	578	85.9		
With partner, not in same household	46	2.8	24	2.5	24	3.6		
No partner	132	8.2	61	6.4	71	10.5		
Educational level (N = 1619)							24.82	<0.001
No qualification	1	0.1	1	0.1	0	0.0		
University entrance qualification	36	2.2	14	1.5	22	3.3		
Certificate of 2 Education (9 yrs of schooling) ^1^	409	25.3	206	21.7	203	30.3		
Certificate of 2 Education (10 yrs of schooling) ^2^	1140	70.4	710	74.8	430	64.1		
Other	33	2.0	17	1.8	16	2.4		
Employment status before pandemic (N = 1619)							20.49	<0.001
Inactive/unemployed	30	1.9	13	1.4	17	2.5		
Maternity leave/parental leave	23	1.4	10	1.1	13	1.9		
Short-term/temporary employment	98	6.1	57	6.0	41	6.1		
Part-time	254	15.7	123	13.0	131	19.5		
Full-time/freelance	1214	75.0	745	78.6	469	69.9		
Household net equivalent income during pandemic (monthly, in EUR, quintiles) (N = 1619)							35.23	<0.001
Up to 850	368	22.7	176	18.6	192	28.6		
850–1000	312	19.3	166	17.5	146	21.8		
1000–1299	314	19.4	201	21.1	113	16.9		
1300–1399	364	22.5	238	25.1	126	18.8		
1400–2750	261	16.1	167	17.6	94	14.0		
Household net equivalent income during pandemic (N = 1275)							7.75	0.021
Less	348	27.3	187	24.6	161	31.3		
Same	874	68.6	537	70.7	337	65.4		
Higher	53	4.2	36	4.7	17	3.3		
SES index (N = 1619)							49.16	<0.001
Low	301	18.6	127	13.4	174	25.9		
Middle	839	51.8	498	52.5	341	50.8		
High	479	29.6	323	34.1	156	23.3		

CSHCN, children with special healthcare needs; SES, socioeconomic status; ^1^ lower secondary school leaving certificate, corresponding roughly to basic high school completion; ^2^ intermediate secondary school leaving certificate, corresponding to a general high school diploma in the United States.

In total, 41.4% caregivers of CSHCN completed the questionnaire, and 71.2% of CSHCN had a screener score ≥3, indicating more complex needs. Moreover, 55.6% of CSHCN were male; their mean age was 9 years (SD = 4.4) (Table 1). In addition, 69.9% of caregivers of CSHCN were working full-time before the pandemic, with a high educational level and an overall high net equivalent income. The SES index was low in 25.9%. During the first round of the survey, the net equivalent income decreased for 31.3% (21.2% missing) of caregivers of CSHCN (Table 1).

The majority of CSHCN attended kindergarten (27.0%), primary school (23.5%), or schools for children with special educational needs (19.8%). In particular, 12.4% of children with less complex needs and 57.1% of children with more complex needs had a physical impairment; 12.7% of children with less complex needs and 60.4% of children with more complex needs had a behavioral impairment (Table 2). Moreover, 49.3% of caregivers of children with more complex needs described the daily nursing care as demanding and 12.2% as very demanding (Table 2).

**Services involved in healthcare provision before the first wave of the pandemic.** Before the pandemic, 43.5% of CSHCN attended regular medical appointments (Table 3). In particular, 38.3% received physiotherapy and 49.0% occupational therapy, speech therapy, or inclusive education (Table 3). Moreover, 24.0% of children were supported by an integration aid in kindergarten or school; 26.8% of CSHCN attended special education facilities (Table 3).

**Associations between complexity of healthcare and children’s disease complexity.** Simple linear regression showed positive associations between the complexity of healthcare and children’s disease complexity (B = 0.57, 95% CI [0.55; 0.59]; *p* < 0.001). Similar results could be shown for both subgroups of disease complexity in both the medical and educational sectors (*p* < 0.001). In a sensitivity analysis incorporating the revised CSHCN Screener score (excluding the three service use items), the results of the linear regression still showed strong evidence for an association between the complexity of healthcare and children’s disease complexity.

**Changes in healthcare provision during the pandemic.** The COVID-19 pandemic led to a reduction in healthcare provision for CSHCN, ranging between 49.0% (doctor’s appointments) and 84.7% (occupational therapy, speech therapy, inclusive education) in the medical sector (Table 4). Moreover, 88.0% (1.9% missing) received less or no support via integration aids in kindergarten or school, and socio-educational family support ceased completely in 65.0% of cases (Table 4).

**Associations of changes in frequency of service provision during the COVID-19 pandemic and associations with disease complexity, psychosocial factors, and SES.** Associations between reductions in care during the first round of the survey and disease complexity could be shown using multiple linear regression analysis (B = 0.50, 95% CI [0.43; 0.57], *p* < 0.001). There was no evidence of an association between reductions in care and SES (B = 0.02, 95% CI [0.03; 0.07], *p* = 0.38) and the psychosocial factors of the children (B = 0.02, 95% CI [0.01; 0.37], *p* = 0.13). Moreover, no association was found between a reduction in care and the psychosocial factors of the caregivers (B = 0.00, 95% CI [0.01; 0.01], *p* = 0.95).

In the sensitivity analysis incorporating the revised CSHCN Screener score, the results of the multiple linear regression still showed strong evidence for an association between a reduction in care during the COVID-19 pandemic and disease complexity (B = 0.94, 95% CI [0.89; 0.99], *p* < 0.001).

## 4. Discussion

To our knowledge, this is the first cross-sectional study that provides data on healthcare provision and reductions in care in the medical, educational, and social sectors in Germany during the first wave of the COVID-19 pandemic in a large sample (n = 1619) of children with and without chronic diseases. The findings reveal a stark picture: up to 88% of children with complex chronic conditions experienced reductions in or the complete disruption of essential healthcare services. These disruptions were noticeable across the medical, therapeutic, educational, and social domains, undermining core components of health and participation for this vulnerable group.

Although children with and without special healthcare needs were comparable regarding core demographic characteristics such as caregiver age and child gender, the families of children with special healthcare needs differed in several socioeconomic aspects. These families were more likely to live in rural areas, to work part-time, and to report lower household incomes and income loss during the pandemic. While these differences did not translate into an association between socioeconomic status and care reduction in our analyses, they highlight the broader structural context in which families of children with special healthcare needs navigate healthcare disruptions. These baseline disparities underscore the importance of designing crisis-resilient care systems that account not only for medical complexity but also for contextual and socioeconomic factors influencing access to care.

**Healthcare provision for CSHCN before and during the COVID-19 pandemic.** Overall, the need for care in CSHCN before the pandemic was very high (Table 3). These circumstances render CSHCN, especially those with complex chronic diseases, particularly vulnerable to changes in healthcare provision [16,17]. The abrupt breakdown of service continuity highlights a critical gap in crisis preparedness within pediatric care systems [5,16,18]. Although Germany has robust healthcare infrastructure under normal conditions, it became evident during the pandemic that these structures are not sufficiently crisis-resilient, particularly for children requiring interdisciplinary and intersectoral care. Their dependency on coordinated services across the health and education sectors made them disproportionately vulnerable. Our results show strong evidence for an association between a reduction in care and children’s disease complexity. This aligns with the international literature indicating that CSHCN often bear the greatest burden when systems are under stress [19,20].

**Potential associations of changes in frequency of service provision with SES and psychosocial factors in families with CSHCN.** Our data showed no evidence for associations between SES and reductions in care in families with CSHCN during the first wave of the COVID-19 pandemic. These results raise the question of whether families with CSHCN in Germany, both those with high and low SES, were equally affected by public health and social measures (PHSM). As our data suggest and international research underlines, access to and utilization of medical care was radically restricted for all population groups during the first wave of the pandemic, which could explain our findings [21,22].

While Geweniger et al. outlined clear associations between complex healthcare needs and children’s psychological well-being, no association between care reductions and the psychological well-being of children and the children’s caregivers could be shown [23]. This may indicate stable family relationships and family support within this study’s population group that enabled them to cope with the abrupt reduction in care in all sub-areas described. As Fogel et al. reported, constant and intimate relationships among family members had significant positive effects on both the family’s and children’s well-being during the first lockdown [24]. However, to evaluate associations between psychological well-being and reductions in care for CSHCN during the course of the pandemic, further research in this area is required.

**Actionable insights for building and reimagining crisis-resilient pediatric care systems.** Our findings highlight substantial disruptions in the care of CSHCN during the first wave of the COVID-19 pandemic in Germany. Children with more complex needs faced significantly greater care reductions, underscoring the system’s fragility under stress. Notably, the reduction in care did not correlate with socioeconomic status or caregiver mental health, suggesting a systemic vulnerability affecting all CSHCN, rather than selective inequality. This highlights the urgent need for resilient and inclusive care infrastructures that do not collapse under emergency conditions. Our results serve as a foundation for the rethinking of pediatric care in three ways:

1. Early identification of vulnerable care pathways—especially for CSHCN with more complex healthcare needs—must guide future emergency planning [25];
2. Cross-sector coordination between health, education, and social services is essential to maintain service continuity during disruptions [26];
3. Digital infrastructure and telehealth solutions should be expanded as fallback systems to prevent total service breakdown [26].

The findings provide several key takeaways for future action.

1. **Resilience through integration.** Crisis-resilient care systems must ensure service continuity for high-need populations even under restrictive conditions. This requires pre-established, integrated care pathways with digital backup solutions (e.g., telemedicine, virtual therapy), sector-bridging coordination, and decentralized emergency care protocols [26].
2. **Institutionalizing family involvement.** Families are experts in their children’s needs. Their voices must be formally integrated into health system design, policy development, and quality assurance. Active participation can improve service relevance and responsiveness, especially in crisis contexts [24].
3. **Research for future readiness.** Although this study found no association between SES and care reductions, future longitudinal research is essential to uncover delayed or cumulative effects, particularly on marginalized families. Future studies should focus on how different social determinants interact with system vulnerabilities during and after public health emergencies.
4. **Mental health requires proactive strategies.** Although no direct association was observed between care reductions and psychological distress in this study, international evidence suggests otherwise [6,27]. The apparent resilience observed in our study may be due to strong family support networks in the sample. Broader, more representative studies are needed to assess the true scope of psychosocial impacts and to inform preventive strategies.
5. **Policy transformation is necessary.** The pandemic must be a turning point. CSHCN and their families should be explicitly included in national emergency response plans. Their access to therapy, education, and support services must be codified as essential—legally protected and operationalized through funded frameworks.

## 5. Limitations

Despite the large sample, the empirical design of this cross-sectional study included a non-representative study group, which limits the generalizability of the study results. Compared to the prevalence of chronic diseases in children and adolescents in the general population (16.2%), CSHCN were over-represented in our study sample [28]. Moreover, the results showed an above-average level of education and SES among the caregivers compared to the total population.

The CSHCN Screener was originally designed as a screening tool that identifies children who both have complex health needs and require additional health service support. Consequently, part of the predictor overlaps with our utilization outcomes, creating a mechanical association. Our sensitivity analyses illustrate that roughly 60% of the screener score reflects service use items (three out of five items). When these are removed, the association between disease complexity and complexity of care/reduction in care still remains, indicating that, even after accounting for measurement overlap, there may be a genuine underlying link. Future work should employ disease complexity measures that are independent of utilization to further investigate this association.

The survey retrospectively assessed the frequency of services provided before the pandemic, which might have led to either over- or underestimating the actual change in service provision during the first wave of the COVID-19 pandemic in Germany. Reasons for reductions in service provision and/or utilization were not elicited.

Regarding statistical evaluation, it must be considered that the percentage of missing values in the respective variables amounted to 15 to 24%. The regression-based multiple imputation approach assumes missing at random. If missingness occurs not at random, bias may persist. Our imputation model omitted higher-order interactions and non-linear terms, which could lead to misspecification. The default predictive mean matching in SPSS and logistic/proportional odds models may be suboptimal for highly skewed distributions, and we performed only visual convergence checks, without formal diagnostics or sensitivity analyses. Hence, our findings involving pooled estimates derived from multiple imputation should be interpreted cautiously [29].

## 6. Conclusions

This study demonstrates that children with complex chronic diseases require multi-professional care in complex care structures and thus were particularly affected by pandemic control measures during the first wave of the COVID-19 pandemic in Germany.

The results indicate high stress and restrictions on families with CSHCN both before and during the pandemic: positive associations between disease complexity and the complexity of healthcare provision identify CSHCN as particularly vulnerable to changes in healthcare provision. A significant reduction in medical–therapeutic care for CSHCN during the COVID-19 pandemic in Germany could be shown.

Potential long-term impacts of this reduction on health, psychological factors, and education in this population group remain unclear and will need future investigation [30]. Additionally, strategies to expand service provision for CSHCN permanently and resiliently will have to be developed and implemented [31]. Clear demands have already been put forward to maintain inpatient, outpatient, rehabilitative, and medical–therapeutic care in crisis without restricting the range of services [32]. As could be shown, CSHCN and their families were severely affected by the pandemic measures and thus should be given particular consideration when reorganizing and reorienting politics and healthcare services to form crisis-resilient frameworks and to contain consequences of the pandemic restrictions [26].

In short, the pandemic acted as a stress test for pediatric care systems and the results revealed significant structural weaknesses. Our data provide clear indicators of where targeted investments, contingency planning, and policy innovation are urgently required to build future-proof, child-centered health systems.

## 7. Key Messages

Children with special healthcare needs are especially vulnerable and must be prioritized in health crisis planning.

CSHCN with more complex needs were shown to be even more vulnerable.

Their care requirements should guide the development of future-resilient service models.

The disruption of multidisciplinary care during the pandemic highlights the need for protected, integrated care pathways.

Longitudinal studies are essential: monitoring the medium- and long-term impacts of pandemic-related care disruptions will help to identify lasting effects and adjust services accordingly.

Inclusive health policy is critical, and family voices must be embedded in health system design, evaluation, and crisis response planning.

Future policies must ensure that pediatric care services—especially for high-need populations—remain uninterrupted during public health emergencies.

## Figures and Tables

**Table 2:** Impairments and healthcare needs of children with less (n = 193) and more complex needs (n = 478).

	Health Conditions of Children					
	Total (N = 671)		Children with Less Complex Needs		Children with More Complex Needs	
	n	%	n	%	n	%
Impairment						
Physical impairment	466	69.6	83	12.4	383	57.1
Behavioral impairment	490	73.0	85	12.7	405	60.4
Impairment in language development	360	53.7	52	7.8	308	45.9
Deficits in communication	188	28.0	9	1.3	179	26.7
Healthcare needs						
Home ventilation	11	1.6	1	0.2	10	1.5
Tube feeding	38	5.7	2	0.3	36	5.4
Daily nursing care						
Very demanding	86	12.8	4	0.6	82	12.2
Demanding	397	59.2	66	9.8	331	49.3
Not demanding	188	28.0	123	18.3	65	9.7

**Table 3:** Healthcare provision in CSHCN (N = 671) before the COVID-19 pandemic in the medical, educational, and social sectors.

			Health Conditions of Children			
	Affirmative Reply		Children with Less Complex Needs		Children with More Complex Needs	
	n	%	n	%	n	%
Healthcare provision in medical sector (N = 671)						
Regularly scheduled doctor’s appointments	292	43.5	50	25.9	242	50.6
Physiotherapy	257	38.3	17	8.8	240	50.2
Occupational therapy/speech therapy/inclusive education	329	49.0	38	19.7	291	60.9
Early intervention	97	14.5	10	5.2	87	18.2
Home care services	51	7.6	1	0.5	50	10.5
Healthcare provision in educational sector (N = 671)						
Nurses in kindergarten/school	46	6.9	3	0.5	43	6.4
Integration aid in kindergarten/school	161	24.0	6	0.9	155	23.1
Special educational care	180	26.8	7	1.0	173	25.8
Healthcare provision in social sector (N = 671)	20	3.0	0	0.0	20	3.0
Social and pedagogical family assistance						

**Table 4:** Healthcare provision during COVID-19 pandemic among CSHCN in medical, educational, and social sectors.

			Health Conditions of Children							
	Total		Children with Less Complex Needs		Children with More Complex Needs					
	n	%	n	%	n	%	Chi *	df	*p*-Value	Exact **
Doctor’s appointments (N = 292)										
Same	147	51.0	31	10.6	116	39.7				
Less/none	143	49.0	18	6.2	125	42.8	3.73	1	0.053	0.038
Physiotherapy (N = 254)										
Same	54	21.3	6	2.4	48	18.9				
Less/none	200	78.7	11	4.3	189	74.4	2.14	1	0.143	0.126
Occupational/speech therapy/inclusive education (N = 326)										
Same	50	15.3	4	1.2	46	14.1				
Less/none	276	84.7	34	10.4	242	74.2	0.77	1	0.381	0.271
Early intervention (N = 95)										
Same	11	11.6	1	1.1	10	10.5				
Less/none	84	66.4	9	9.5	75	79.0	0.03	1	0.869	0.674
Home care services (N = 51)										
Same	20	39.2	0	0.0	20	39.2				
Less/none	31	60.8	0	0.0	31	60.8	0.66	1	0.417	0.608
Nurses in kindergarten/school (N = 44)										
Same	8	18.2	1	2.3	7	15.9				
Less/none	36	81.8	2	4.6	34	77.3	0.50	1	0.481	0.461
Integration aid (N = 158)										
Same	19	12.0	1	0.6	18	11.4				
Less/none	139	88.0	4	2.5	135	85.4	0.31	1	0.580	0.478
Special educational care										
Emergency care (N = 88)	88	100.0	35	39.8	53	60.2	0.70	1	0.403	0.453
Online support (N = 380)	380	100.0	133	35.0	247	65.0	5.24	1	0.073	0.070
Socio-educational family support (N = 20)										
Same	7	35.0	0	0.0	7	35.0	-	-	-	-
Less/none	13	65.0	0	0.0	13	65.0				

* Chi-squared test. ** Fisher’s exact test.

## Data Availability

The original contributions presented in this study are included in the article. Further inquiries can be directed to the corresponding author.

## Abbreviations

The following abbreviations are used in this manuscript:

WHO	World Health Organization
CSHCN	Children with special healthcare needs
SES	Socioeconomic status
SDQ	Strengths and Difficulties Questionnaire

## References

[1] Calvano C., Engelke L., Bella J.D., Kindermann J., Renneberg B., Winter S.M. (2021). Families in the COVID-19 pandemic: Parental stress, parent mental health and the occurrence of adverse childhood experiences—Results of a representative survey in Germany. Eur. Child Adolesc. Psychiatry.

[2] World Health Organization (2020). Pulse Survey on Continuity of Essential Health Services During the COVID-19 Pandemic—Interim Report 2020.

[3] European Union (2022). Health at a Glance: Europe 2022. State of Health in the EU Cycle.

[4] Schilling J., Tolksdorf K., Marquis A., Faber M., Pfoch T., Buda S., Haas W., Schuler E., Altmann D., Grote U. (2021). Die verschiedenen Phasen der COVID-19-Pandemie in Deutschland: Eine deskriptive Analyse von Januar 2020 bis Februar 2021. Bundesgesundheitsblatt-Gesundheitsforschung-Gesundheitsschutz.

[5] Wang Z., Tang K. (2020). Combating COVID-19: Health equity matters. Nat. Med..

[6] Ravens-Sieberer U., Kaman A., Otto C., Adedeji A., Napp A.K., Becker M., Blanck-Stellmacher U., Löffler C., Schlack R., Hölling H. (2021). Seelische Gesundheit und psychische Belastungen von Kindern und Jugendlichen in der ersten Welle der COVID-19-Pandemie—Ergebnisse der COPSY-Studie. Bundesgesundheitsblatt Gesundheitsforschung Gesundheitsschutz.

[7] Cohen S., Toly V., Lerret S., Sawin K. (2023). The impact of COVID-19 on systems of care for children and youth with special health care needs. J. Pediatr. Health Care.

[8] Diskin C., Buchanan F., Cohen E., Dewan T., Diaczun T., Gordon M., Lee E., MooreHepburn C., Major N., Orkin J. (2022). The impact of the COVID-19 pandemic on children with medical complexity. BMC Pediatr..

[9] Bethell C., Read D., Stein R., Blumberg S., Wells N., Newacheck P. (2002). Identifying children with special health care needs: Development and evaluation of a short screening instrument. Ambul. Pediatr..

[10] Bethell C.D., Blumberg S.J., Stein R.E.K., Strickland B., Robertson J., Newacheck P.W. (2015). Taking stock of the CSHCN Screener: A review of common questions and current reflections. Acad. Pediatr..

[11] Woerner W., Becker A., Friedrich C., Rothenberger A., Klasen H., Goodman R. (2002). Normierung und Evaluation der deutschen Elternversion des Strengths and Difficulties Questionnaire (SDQ): Ergebnisse einer repräsentativen Felderhebung. Z. Kinder-Jugendpsychiatrie Psychother..

[12] Rothenberger A., Becker A., Erhart M., Wille N., Ravens-Sieberer U. (2008). Psychometric properties of the parent strengths and difficulties questionnaire in the general population of German children and adolescents: Results of the BELLA study. Eur. Child. Adolesc. Psychiatry.

[13] Croft S., Stride C., Maughan B., Rowe R. (2015). Validity of the Strengths and Difficulties Questionnaire in Preschool-Aged Children. Pediatrics.

[14] Topp C.W., Østergaard S.D., Søndergaard S., Bech P. (2015). The WHO-5 well-being index: A systematic review of the Literature. Psychother. Psychosom..

[15] Lampert T., Kroll L., Müters S., Stolzenberg H. (2013). Messung des sozioökonomischen Status in der Studie zur Gesundheit Erwachsener in Deutschland (DEGS1). Bundesgesundheitsblatt-Gesundheitsforschung-Gesundheitsschutz.

[16] Stork T., Dilger C., Schindler V., Leher A., Böhme M. (2020). Kindergesundheitsbericht Baden-Württemberg 2020.

[17] Kurth B.M., Schmich P., Kuhnert R., Robert Koch-Institut, Abteilung Für Epidemiologie Und Gesundheitsmonitoring (2019). KiGGS Welle 2—Studie zur Gesundheit von Kindern und Jugendlichen in Deutschland. RKI Robert Koch Institute. https://www.rki.de/DE/Themen/Nichtuebertragbare-Krankheiten/Studien-und-Surveillance/Studien/KiGGS/kiggs_start_inhalt.html.

[18] Feral-Pierssens A.L., Claret P.G., Chouihed T. (2020). Collateral damage of the COVID-19 outbreak: Expression of concern. Eur. J. Emerg. Med..

[19] Evliyaoğlu O. (2020). Children with chronic disease and COVID-19. Türk. Pediatri. Arş..

[20] Gigli K.H., Graaf G. (2022). Changes in use and access to care for children and youth with special health care needs during the COVID-19 pandemic. J. Pediatr. Health Care.

[21] Chanchlani N., Buchanan F., Gill P.J. (2020). Addressing the indirect effects of COVID-19 on the health of children and young people. CMAJ.

[22] UNICEF Deutschland (2021). Corona ist Größte Krise für Kinder seit Gründung.

[23] Geweniger A., Barth M., Haddad A.D., Högl H., Insan S., Mund A., Langer T. (2022). Impact of the COVID-19 pandemic on mental health outcomes of healthy children, children with special health care needs and their caregivers–results of a cross-sectional study. Front. Pediatr..

[24] Fogel Y., Sela Y., Hen-Herbst L. (2022). Coping strategies of families and their relationships with family quality of life during COVID-19 pandemic. PLoS ONE.

[25] Corona-ExpertInnenrat der Bundesregierung (2022). 7. Stellungnahme: Zur Notwendigkeit einer Prioritären Berücksichtigung des Kindeswohls in der Pandemie.

[26] Hefferon C., Taylor C., Bennett D., Falconer C., Campbell M., Williams J.G., Schwartz D., Kipping R., Taylor-Robinson D. (2021). Priorities for the child public health response to the COVID-19 pandemic recovery in England. Arch. Dis. Child..

[27] Lignou S., Greenwood J., Sheehan M., Wolfe I. (2022). Changes in healthcare provision during COVID-19 and their impact on children with chronic illness: A scoping review. Inq. J. Med. Care Organ. Provis. Financ..

[28] Mauz E., Schmitz R., Poethko-Müller C. (2017). Kinder und Jugendliche mit besonderem Versorgungsbedarf im Follow-up: Ergebnisse der KiGGS-Studie 2003–2012. J. Health Monit..

[29] Marchenko Y., Reiter J. (2009). Improved Degrees of Freedom for Multivariate Significance Tests Obtained from Multiply Imputed, Small-Sample Data. Stata J. Promot. Commun. Stat. Stata.

[30] Kohns Vasconcelos M., Weil K., Vesterling-Hörner D., Klemm M., el Scheich T., Renk H., Remke K., Bosse H.M. (2021). Paediatric primary care in Germany during the early COVID-19 pandemic: The calm before the storm. Fam. Med. Community Health.

[31] Die Bundesregierung (2021). Kindergesundheit: Wie Kinder unter der Pandemie leiden.

[32] Bundesministerium für Gesundheit (2017). Allianz für Gesundheitskompetenz.

